# Retinal Vascular Changes in Alzheimer's Dementia and Mild Cognitive Impairment: A Pilot Study Using Ultra-Widefield Imaging

**DOI:** 10.1167/tvst.12.1.13

**Published:** 2023-01-09

**Authors:** Emma Pead, Atalie C. Thompson, Dilraj S. Grewal, Sarah McGrory, Cason B. Robbins, Justin P. Ma, Kim G. Johnson, Andy J. Liu, Charlene Hamid, Emanuele Trucco, Craig W. Ritchie, Graciela Muniz, Imre Lengyel, Baljean Dhillon, Sharon Fekrat, Tom MacGillivray

**Affiliations:** 1 VAMPIRE Project, Centre for Clinical Brain Sciences, The University of Edinburgh, Edinburgh, UK; 2 Department of Ophthalmology, Duke University School of Medicine, Durham, NC, USA; 3 Department of Neurology, Duke University School of Medicine, Durham, NC, USA; 4 Edinburgh Clinical Research Facility, The University of Edinburgh, Edinburgh, UK; 5 VAMPIRE Project, Computer Vision and Image Processing, Computing (SSE), The University of Dundee, Dundee, UK; 6 Edinburgh Dementia Prevention, The University of Edinburgh, Edinburgh, UK; 7 Department of Social Medicine, Ohio University, Athens, OH, USA; 8 The Welcome-Wolfson Institute for Experimental Medicine, School of Medicine Dentistry and Biomedical Science, Queen's University Belfast, Belfast, UK; 9 Princess Alexandra Eye Pavilion, NHS Lothian, Edinburgh, UK

**Keywords:** Alzheimer's dementia, Alzheimer's disease, mild cognitive impairment, microvascular, normal cognition, ultra-widefield retinal imaging, peripheral retina, retinal image analysis, retinal biomarkers

## Abstract

**Purpose:**

Retinal microvascular abnormalities measured on retinal images are a potential source of prognostic biomarkers of vascular changes in the neurodegenerating brain. We assessed the presence of these abnormalities in Alzheimer's dementia and mild cognitive impairment (MCI) using ultra-widefield (UWF) retinal imaging.

**Methods:**

UWF images from 103 participants (28 with Alzheimer's dementia, 30 with MCI, and 45 with normal cognition) underwent analysis to quantify measures of retinal vascular branching complexity, width, and tortuosity.

**Results:**

Participants with Alzheimer's dementia displayed increased vessel branching in the midperipheral retina and increased arteriolar thinning. Participants with MCI displayed increased rates of arteriolar and venular thinning and a trend for decreased vessel branching.

**Conclusions:**

Statistically significant differences in the retinal vasculature in peripheral regions of the retina were observed among the distinct cognitive stages. However, larger studies are required to establish the clinical importance of our findings. UWF imaging may be a promising modality to assess a larger view of the retinal vasculature to uncover retinal changes in Alzheimer’s disease.

**Translational Relevance:**

This pilot work reports an investigation into which retinal vasculature measurements may be useful surrogate measures of cognitive decline, as well as technical developments (e.g., measurement standardization), that are first required to establish their recommended use and translational potential.

## Introduction

The pathophysiological process of Alzheimer's disease begins decades before the symptoms of dementia emerge, and the progression spectrum is currently described in three stages.^1^ Preclinical Alzheimer's disease describes the stage where abnormal accumulation of amyloid beta (Aβ) plaque deposition and neurofibrillary tau tangles in the brain may already be in progress, with no significant clinical symptoms.^1^ Mild cognitive impairment (MCI), the second stage, is a clinical condition described by a measurable decline in cognitive abilities, notably without loss of functional independence, which clinically distinguishes MCI from preclinical Alzheimer's disease.^2^ Alzheimer's dementia is the final stage, with clinical presentation of symptoms that fit a pattern of memory dysfunction and loss of functional independence in multiple cognitive domains.^1^ Approximately 35% of people with MCI will progress to Alzheimer's dementia within 5 years of diagnosis^3^; however, some individuals with Alzheimer's disease pathophysiology may never become symptomatic in their lifetime. Although changes in cerebrospinal fluid, protein biomarkers, and positron emission tomography have the greatest utility in identifying the different phases of the Alzheimer's disease spectrum, these methods are invasive and expensive. Effective and cost-efficient biomarkers to assist in the detection and monitoring of Alzheimer's disease are therefore highly desirable to aid in early interventions to prevent or delay dementia onset.

Due to the homology between the retina and the brain,^4^ abnormalities in the eye are thought to reflect deteriorating microvascular health, Aβ plaque deposition, and neuroaxonal degeneration in the brain.^5^^,^^6^ With increasing recognition given to the contribution of the health of small cerebral vessels in the pathogenesis of Alzheimer's disease,^7^^,^^8^ the prospect of utilizing the retinal vasculature as surrogate for the health of the brain's microvasculature is of particular interest.^9^

Ultra-widefield (UWF) imaging captures larger views of the retina than are possible with standard fundus photography (an angular field of view of up to 200° compared to conventional 30°–45° fundus photography) and is achieved with a scanning laser ophthalmoscope (SLO). This technology enables a more comprehensive view of the retina to be examined for vascular signs pertaining to Alzheimer's dementia and MCI, particularly in peripheral areas that are inaccessible to other instruments and therefore rarely explored.^10^ A recently reported use of UWF imaging demonstrated changes in the vasculature in Alzheimer's dementia beyond the central retina or posterior pole during a 2-year period, suggesting that observing for pathological changes in the peripheral retina might have value in monitoring the progression of neurodegenerative disease.^6^

In this study, we investigated whether associations exist between retinal changes measured in both central and peripheral regions of the retina using UWF imaging in people with a diagnosis of Alzheimer's dementia or MCI by comparing to a group of individuals with normal cognition. We report retinal vascular characteristics that might translate to candidate biomarkers for Alzheimer's disease and its progression.

## Materials and Methods

### Participant Recruitment

The Institutional Review Board for Human Research at the Duke University School of Medicine (Durham, NC) approved the procedures for the study, which followed the tenets of the Declaration of Helsinki (Clinicaltrials.gov identifier NCT 03233646). Written informed consent was obtained from study participants or an authorized representative. People with Alzheimer's dementia and MCI who were 50 years or older were recruited from the Duke Memory Disorders Clinic. Evaluation and diagnosis of Alzheimer's dementia and MCI were performed by an experienced neurologist with expertise in memory disorders (AJL) based on clinical history and cognitive testing in accordance with diagnostic guidelines and recommendations of the National Institute on Aging–Alzheimer's Association.^11^^,^^12^ Individuals with normal cognition who were 50 years or older and without subjective memory symptoms were recruited from spouses of patients in attendance at the Duke Memory Disorders Clinic or from the Bryan Alzheimer's Disease Research Center registry for control participants. To avoid confounding any relationship between retinal changes and Alzheimer's dementia, MCI, or individuals with normal cognition, exclusion criteria to recruitment included non-Alzheimer's dementia, diabetes, uncontrolled hypertension, demyelinating disorders, glaucoma, age-related macular degeneration, prior intraocular surgery other than cataract surgery, and visual acuity worse than 20/40 on the day of image acquisition. All participants underwent a Mini-Mental State Examination (MMSE)^13^ to assess cognitive function on the same day as image acquisition. All participants had normal optical coherence tomography imaging, and 90% were Caucasian American.

### Image Acquisition and Image Analysis

UWF images were acquired in the Duke University Memory Disorders Clinic without pharmacologic mydriasis using a California SLO (Optos, Inc., Marlborough, MA). Red (633 nm) and green (532 nm) lasers in the instrument are reflected off a concave elliptical mirror that directs the beams to regions of the retina inaccessible with conventional fundus photography. The images from the device undergo stereographic projection to take into account curvature at the back of the eye and enable measurements of distance (in millimeters) on the retina to be obtained.^14^ Proprietary software from the manufacturer projects the image (fovea-centered; on-axis resolution of approximately 15 µm; TIFF format; dimensions of 4000 × 4000 pixels) along with an estimation of the reliability of the projection process. As recommended by the manufacturer, images that have suboptimal projection were deemed unsuitable for obtaining distance measures; however, they were suitable for analysis of branching complexity that is not dependent on accurate correction of peripheral distortion.^15^

Measurements of the retinal vasculature were performed using custom software (ultra-widefield version of Vasculature Assessment and Measurement Platform for Images of the Retina [VAMPIRE-UWF]; Universities of Edinburgh and Dundee, UK), specially designed for analyzing UWF images.^16^^,^^17^ First, vessels are automatically segmented and skeletonized by VAMPIRE, which can be manually corrected by the operator to remove erroneous detections that can occur due to the presence of drusen, reflections (due to suboptimal patient positioning), or choroidal vessels (due to blonde fundus). The software is then used to return measurements that characterize the state or health of the vasculature (as described below).

#### Fractal Analysis

**Figure 1.**
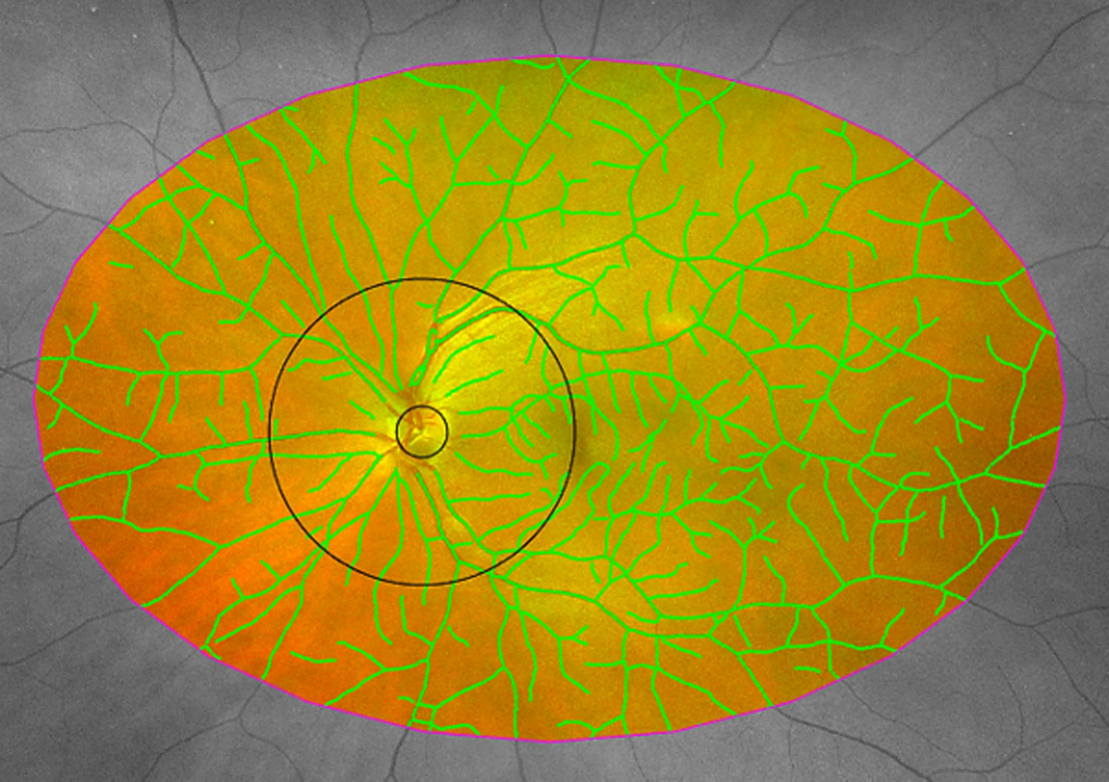
Measurement of vessel network complexity from the skeleton of the automatically segmented vessels in UWF imaging in the standardized (between *black* and *magenta outlines*), posterior (*black*), and midperiphery (*black* to *magenta*) ROIs.

All UWF images in this study underwent multifractal analysis to quantify fractal dimension (FD) of the vessel branching patterns contained therein. FD is a unitless measure that quantifies how a repeating pattern (such as vascular branching in the retina) fills the space in which it is contained. The multifractal approach uses the generalized sandbox method and has been described elsewhere.^18^ In brief, multiple FDs are measured at a selection of random points. Multifractal dimensions are more versatile in describing geometrical properties of patterns such as the retinal vasculature, which displays multifractal properties. We computed FD as the multifractal dimension *D*_0_, as this has previously been reported to be a sensitive marker of small vascular changes.^19^ Higher FD values indicate a pattern that is more space filling (often described as more complex). FD is influenced by the space or region of interest (ROI) in which it is measured; for example, the FD of a vessel branching pattern will increase if the space in which it is contained decreases (i.e., it fills the smaller space more).^20^ We therefore standardized the region of the retina for which the FD of a vessel branching pattern is measured so that it is always calculated for the same area within each image. To construct this standardized ROI, a trained operator manually drew a ROI onto each UWF image to exclude artifacts in the image, such as eyelashes and eyelids, that can obscure parts of the retina and interfere with analysis. The standardized ROI was then defined as the intersection of all manually drawn ROIs for the entire dataset (right and left eyes independently) (Supplementary Fig. S1). Furthermore, to investigate regional changes in the complexity of the branching pattern, we defined a ROI that is an annulus centered on the optic disc that extends three optic disc diameters away from the optic disc boundary^21^ (denoted as posterior) and a ROI between posterior and standardized (denoted as midperiphery) (Fig. 1). FD was computed^18^ within the three regions to quantify vascular branching complexity.

#### Vessel Width Gradient and Tortuosity Analysis

To quantify how a vessel narrows or tapers the farther away it gets from the optic nerve, we calculate a parameter referred to as the width gradient (WG). This is the rate of change in vessel width measured by computing the gradient of the robust regression line^22^ that fits measurements of vessel width (microns) against distance along the path (millimeters).^6^ In addition, tortuosity (Tort) is a unitless measure of curvature (i.e., deviation from a straight line) of a vessel along its path, and we computed it using methods from Annunziata et al.^23^ As the measurements of WG and tortuosity require an accurate correction for peripheral distortion, only images with a satisfactory projection were included in this analysis.

**Figure 2.**
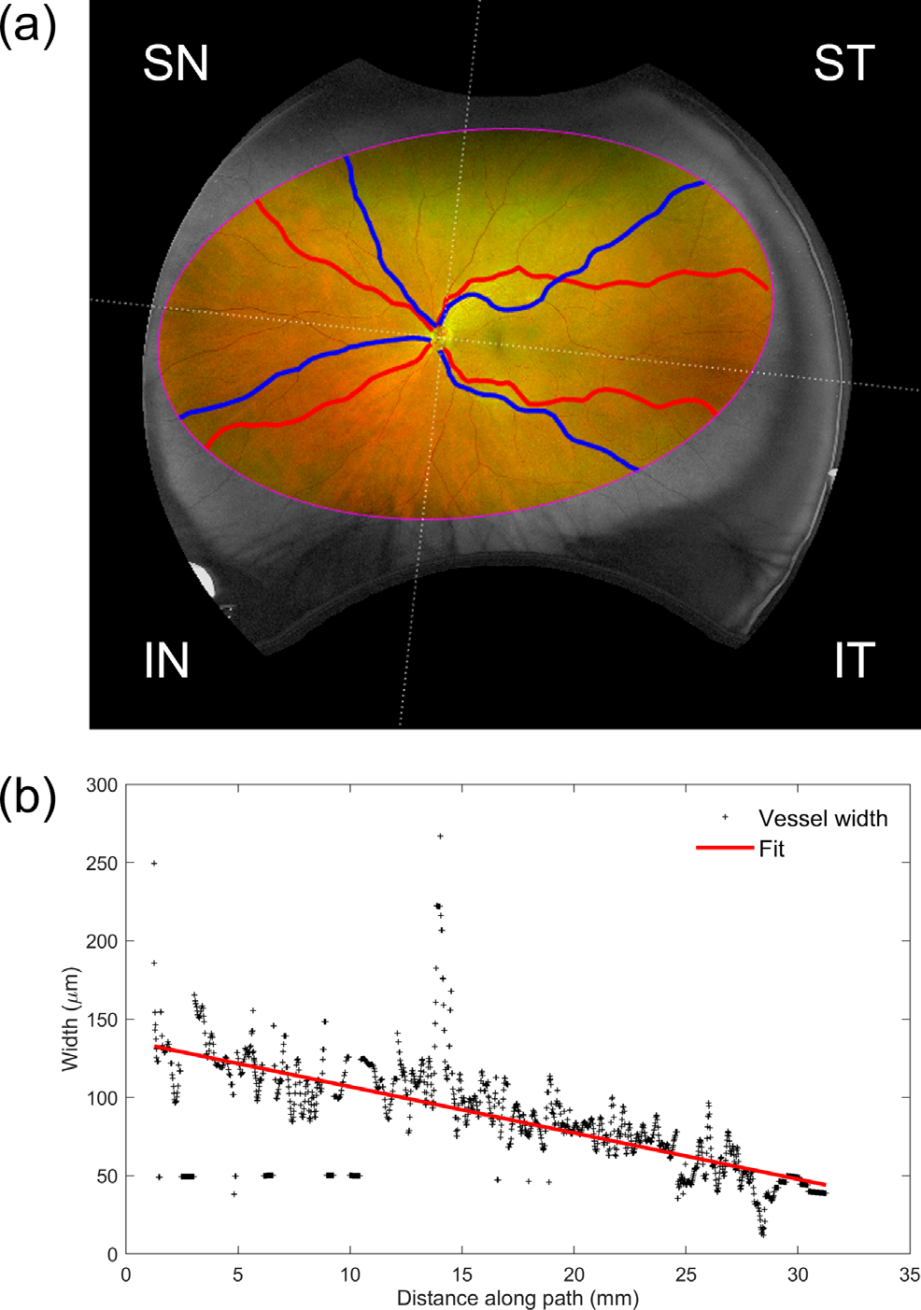
Measurement of vessel width and tortuosity on UWF imaging. (a) Measurements are performed on the prominent arteriolar (*red*) and venular (*blue*) paths within each of the four retinal quadrants (ST, superotemporal; SN, superonasal; IT, inferotemporal; IN, inferonasal) within the manual ROI (*magenta*). (b) Measuring the average rate of change of vessel width, with an example showing the ST venule in (a). Width measurements (µm) are plotted against distance along the vessel path (mm), and the line of best fit is calculated by robust regression to yield the width gradient (µm/mm).

To investigate regional changes in WG and tortuosity, images were divided into quadrants. Quadrants were defined using a horizontal line crossing the center of the manually annotated optic disc and the center of the fovea and another perpendicular to this line crossing the optic disc center. This generated superotemporal, superonasal, inferotemporal, and inferonasal quadrants. The operator manually identified the most prominent, unbroken arteriolar and venular paths segmented by the software in each quadrant. The selection criteria for the paths were that they must be the largest and longest vessel in that quadrant and that the vessels have not been obscured or fragmented by poor image quality (i.e., low contrast making a vessel difficult for the software to distinguish from background tissue, pathology, or artifact) (Fig. 2a). Vessel widths and curvature were automatically computed by VAMPIRE for the selected vessel paths to obtain the WG and Tort measures.

### Statistical Analysis

Statistical analysis was conducted in R 3.6.0 (R Foundation for Statistical Computing, Vienna, Austria). Because the participant ages and their MMSE scores were non-normally distributed, participant mean age and MMSE were compared using a Kruskal–Wallis test. To assess which independent variable differed, Dunn–Šidák post hoc tests were conducted. A χ^2^ test was performed to compare categorical variables between groups (sex and eye).

The comparison of retinal vessel parameters for participant groups was performed using generalized estimating equations (GEEs), using the R package geepack,^24^ to account for correlation between eyes of individuals with an exchangeable working covariance matrix and correction for age and sex. As tortuosity measurements demonstrated a positively skewed distribution, values were normalized using log transformation before GEE analysis. Participant group was the independent variable, and vessel parameter was the outcome. In GEE modeling, the β-coefficient is interpreted as one unit increase or decrease in the outcome. Missing values were imputed to the mean of the group. As GEEs are sensitive to outliers,^25^ extreme values were imputed to the mean of the group. The significance level was evaluated at alpha = 0.05.

## Results

Two hundred and twenty-nine images were captured from 34 patients with a diagnosis of Alzheimer's dementia (31 right eyes, 30 left eyes), 31 patients with MCI (29 right eyes, 25 left eyes), and 60 participants deemed to have normal cognition (58 right eyes, 56 left eyes). We excluded 64 images (18 Alzheimer's dementia, 8 MCI, and 35 normal cognition eyes) due to the presence of eyelashes and eyelids obstructing the view of retinal vasculature and/or low-quality vessel segmentation. The 165 images that were considered suitable for analysis corresponded to 28 participants with Alzheimer's dementia (43 eyes), 30 participants with MCI (45 eyes), and 45 normal cognition participants (77 eyes). Characteristics of these participants are shown in Table 1. The Alzheimer's dementia group was slightly older than the normal cognition participants (73.1 vs. 67.5 years; *P* = 0.013). Participants with Alzheimer's dementia and MCI had MMSE scores indicative of their diagnoses that significantly differed between the groups.

**Table 1.** Participant Characteristics by Group

Characteristic	AD (*n* = 28)	MCI (*n* = 30)	Normal Cognition (*n* = 45)	*P*	Post Hoc Comparisons	Post Hoc *P*
Right eye, *n* (%)	23 (53.5)	25 (55.6)	41 (53.2)	0.98	—	—
Female, *n* (%)	19 (67.8)	16 (53.3)	34 (73.9)	0.26	—	—
Age (y), mean ± SD	73.1 ± 8.8	70.2 ± 0.5	67.5 ± 8.2	**0** **.016**	Normal cognition vs. AD	**0** **.013**
MMSE score, mean ± SD	19.9 ± 5.4	26.3 ± 2.8	29.1 ± 1.2	**<** **0** **.001**	Normal cognition vs. MCI	**0** **.009**
					Normal cognition vs. AD	**<** **0** **.001**
					MCI vs. AD	**0** **.001**

The χ^2^ test was used for sex and eye; the Kruskal–Wallis test was used for MMSE and age. AD, Alzheimer's dementia; MCI, mild cognitive impairment; MMSE = Mini-Mental State Examination.

For retinal vessel WG and tortuosity analysis, we excluded images that did not have an accurate stereographic projection. This corresponded to five participants with Alzheimer's dementia, six with MCI, and eight normal cognition participants. Characteristics for the remaining participants are detailed in Table 2. As above, participants with Alzheimer's dementia were significantly older than normal cognition participants (71.8 vs. 66.5 years; *P* = 0.004), and those with Alzheimer's dementia and MCI had MMSE scores indicative of their diagnoses that significantly differed among participant groups.

**Table 2.** Participant Characteristics for Images That Underwent Retinal Vessel WG and Tortuosity Analysis

Characteristic	AD (*n* = 23)	MCI (*n* = 24)	Normal Cognition (*n* = 37)	*P*	Post Hoc Comparisons	Post Hoc *P*
Right eye, *n* (%)	19 (57.6)	17 (53.1)	34 (58.6)	0.88	—	—
Female, *n* (%)	15 (65.2)	13 (72.2)	28 (75.7)	0.14	—	—
Age (y), mean ± SD	71.8 ± 6.6	70.6 ± 9.8	66.5 ± 7.8	**0** **.004**	Normal cognition vs. AD	**0** **.004**
MMSE score, mean ± SD	19.0 ± 5.5	26.5 ± 3.0	29.1 ± 1.1	**<** **0** **.001**	Normal cognition vs. MCI	**0** **.035**
					Normal cognition vs. AD	**<** **0** **.001**
					MCI vs. AD	**<.001**

The χ^2^ test was used for sex and eye; the Kruskal–Wallis test was used for MMSE and age.

### Fractal Analysis

Participants shown in Table 1 underwent fractal analysis to assess vascular branching complexity. FD measurements suggested that retinal vascular branching was sparser in the MCI participants (regardless of the ROI), whereas the participants with Alzheimer's dementia displayed a more complex arrangement of vessels compared to MCI and normal cognition participants (Table 3). Further analysis with GEE models assessing the relationship between FD measures within each ROI and diagnosis, adjusted for age, showed no evidence of group differences among Alzheimer's dementia, MCI, and normal cognition participants within the standardized and posterior ROI. However, participants with Alzheimer's dementia exhibited significantly higher FD in the midperiphery ROI, indicating a more complex branching complexity compared to normal cognition participants (β = 0.01; *P* < 0.001) and to those with MCI (β = 0.02; *P* < 0.001).

**Table 3.** Comparison of FD Measures Within the Three ROIs Defined on UWF Imaging by GEE Analysis

				AD vs. Normal Cognition		MCI vs. Normal Cognition		AD vs. MCI	
ROI	AD Mean ± SD	MCI Mean ± SD	Normal Cognition Mean ± SD	β (95% CI)	*P*	β (95% CI)	*P*	β (95% CI)	*P*
Standardized	1.529 ± 0.026	1.521 ± 0.029	1.527 ± 0.025	0.003 (−0.003 to 0.008)	0.31	−0.007 (−0.02 to 0.003)	0.19	0.01 (0.0001–0.02)	0.05
Posterior	1.450 ± 0.018	1.44 ± 0.027	1.447 ± 0.021	0.002 (−0.002 to 0.006)	0.34	−0.006 (−0.02 to 0.004)	0.25	0.01 (−0.001 to 0.02)	0.09
Midperiphery	1.434 ± 0.028	1.410 ± 0.019	1.415 ± 0.025	0.01 (0.005–0.02)	**<0.001**	−0.005 (−0.01 to 0.004)	0.31	0.02 (0.01–0.04)	**<0.001**

Standardized is the FD of the retinal vasculature measured in the standardized ROI; posterior is the FD of the retinal vasculature measured within an annulus extending two optic disc diameters away from the optic disc boundary; midperiphery is the FD of the retinal vasculature measured in the standardized ROI excluding zone C. The model was adjusted for age and sex. Bolded values are statistically significant.

### Vessel WG and Tortuosity

Participants detailed in Table 2 were assessed for changes in vessel WG and tortuosity. WG measures in general indicated increased rates of arteriole and venule thinning in participants with Alzheimer's dementia and MCI compared to individuals with normal cognition (Table 4). Analysis with GEE models showed this trend to be significant in Alzheimer's dementia (β = −0.7; *P* < 0.001) and MCI (β = −0.6; *P* < 0.02) when compared to normal cognition participants. The mean venular WG indicated a higher rate of venular thinning in Alzheimer's dementia and MCI. This trend was significant when comparing MCI to normal cognition participants (β = −0.5; *P* < 0.01), but no evidence of a significant difference was found when comparing Alzheimer's dementia to normal cognition participants (β = −0.1; *P* = 0.45). Further examination by quadrant in Alzheimer's dementia showed a significant difference in the superotemporal (β = −0.9; *P* = 0.02) and inferotemporal (β = −0.9; *P* < 0.001) quadrants when compared to normal cognition participants. With MCI, a significantly lower arteriole WG was observed in the inferonasal quadrant compared to normal cognition participants (β = −1.0; *P* = 0.04). A significant increase in venular WG in the inferotemporal quadrant was observed in participants with Alzheimer's dementia compared to normal cognition participants (β = −0.7; *P* = 0.03) (Table 4).

**Table 4.** Comparison of UWF Measures of WG Between Groups by GEE Analysis

					AD vs. Normal Cognition		MCI vs. Normal Cognition		AD vs. MCI	
Quadrant		AD WG (µm/mm) (SD)	MCI WG (µm/mm) (SD)	Normal Cognition WG (µm/mm) (SD)	β (95% CI)	*P*	β (95% CI)	*P*	β (95% CI)	*P*
All	A	−3.8 (0.8)	−3.7 (1.1)	−3.0 (0.7)	−0.7 (−1.1 to −0.4)	**<** **0** **.001**	−0.6 (−1.2 to −0.1)	**0** **.02**	−0.1 (−0.7 to 0.4)	0.64
All	V	−4.1 (0.5)	−4.4 (0.8)	−3.9 (0.9)	−0.1 (−0.4 to 0.2)	0.45	−0.5 (−0.9 to −0.1)	**0** **.01**	0.3 (−0.06 to 0.6)	0.11
ST	A	−4.3 (1.7)	−4.1 (1.5)	−3.4 (1.4)	−0.9 (−1.6 to −0.2)	**0** **.02**	−0.6 (−1.2 to −0.0001)	0.05	−0.3 (−1.1 to 0.5)	0.50
ST	V	−4.7 (1.1)	−5.0 (1.6)	−4.4 (1.1)	−0.2 (−0.6 to 0.3)	0.45	−0.5 (−1.2 to 0.1)	0.11	0.3 (−0.4 to 0.9)	0.39
IT	A	−3.7 (1.3)	−3.0 (1.5)	−2.7 (1.2)	−0.9 (−1.4 to −0.3)	**<** **0** **.001**	−0.3 (−1.1 to 0.4)	0.37	−0.6 (−1.4 to 0.1)	0.12
IT	V	−4.6 (1.3)	−4.2 (0.9)	−3.9 (1.7)	−0.7 (−1.3 to −0.07)	**0** **.03**	−0.3 (−0.8 to 0.3)	0.36	−0.5 (−1.0 to 0.06)	0.08
IN	A	−3.9 (1.7)	−4.5 (2.4)	−3.3 (1.4)	−0.4 (−1.2 to 0.3)	0.24	−1.0 (−3.0 to −0.03)	**0** **.04**	0.4 (−0.6 to 1.3)	0.43
IN	V	−3.6 (1.2)	−4.4 (1.1)	−4.0 (1.4)	0.4 (−0.1 to 1.0)	0.14	−0.3 (−0.8 to 0.2)	0.25	0.7 (0.1 to 1.3)	**0** **.01**
SN	A	−3.2 (1.2)	−2.9 (1.6)	−2.5 (1.4)	−0.5 (−1.1 to 0.01)	0.05	−0.3 (−1.0 to 0.4)	0.39	−0.3 (−0.9 to 0.3)	0.41
SN	V	−3.3 (1.1)	−3.9 (2.0)	−3.1 (1.4)	−0.1 (−0.7 to 0.4)	0.58	−0.6 (−1.5 to 0.2)	0.12	0.5 (−0.3 to 1.3)	0.26

The model was adjusted for age and sex. Bolded values are statistically significant. A, arteriolar; V, venular.

Mean arteriole tortuosity was higher in participants with MCI (−4.7 ± 0.4) compared to participants with Alzheimer's dementia (−4.6 ± 0.2) and normal cognition participants (−4.6 ± 0.3). Mean venular tortuosity was similar in participants with MCI (−4.7 ± 0.2) and normal cognition participants (−4.7 ± 0.3) but lower (i.e., straighter vessels with fewer bends) in participants with Alzheimer's dementia. Further analysis with GEE models and by quadrant showed a significantly higher superonasal arteriolar tortuosity in Alzheimer's dementia (β = −0.2; *P* = 0.04) and MCI (β = −0.5; *P* = 0.009) when compared to normal cognition participants. No significant differences in tortuosity values were evident in other quadrants (Table 5).

**Table 5.** Comparison of UWF Measures of Tortuosity Between Groups by GEEs

					AD vs. Normal Cognition		MCI vs. Normal Cognition		AD vs. MCI	
Quadrant		AD Mean ± SD	MCI Mean ± SD	Normal Cognition Mean ± SD	β (95% CI)	*P*	β (95% CI)	*P*	β (95% CI)	*P*
All	A	−4.6 (0.2)	−4.7 (0.4)	−4.6 (0.3)	−0.02 (−0.1 to 0.1)	0.76	−0.1 (−0.3 to 0.03)	0.09	0.1 (−0.02 to 0.3)	0.08
All	V	−4.6 (0.2)	−4.7 (0.2)	−4.7 (0.3)	−0.001 (−0.1 to 0.1)	0.98	−0.01 (−0.1 to 0.1)	0.85	0.006 (−0.1 to 0.1)	0.90
ST	A	−4.4 (0.3)	−4.4 (0.4)	−4.4 (0.4)	0.007 (−0.2 to 0.2)	0.94	−0.06 (−0.3 to 0.1)	0.56	0.09 (−0.1 to 0.3)	0.39
ST	V	−4.5 (0.3)	−4.4 (0.3)	−4.5 (0.3)	0.06 (−0.06 to 0.2)	0.32	0.1 (−0.03 to 0.3)	0.12	−0.06 (−0.2 to 0.1)	0.43
IT	A	−4.2 (0.4)	−4.4 (0.3)	−4.2 (0.5)	−0.02 (−0.2 to 0.2)	0.84	−0.1 (−0.3 to 0.04)	0.13	0.1 (−0.09 to 0.3)	0.29
IT	V	−4.5 (0.2)	−4.4 (0.2)	−4.5 (0.2)	−0.006 (−0.1 to 0.1)	0.91	0.02 (−0.08 to 0.1)	0.65	−0.069 (−0.2 to 0.04)	0.23
IN	A	−4.8 (0.4)	−4.9 (0.4)	−5.0 (0.6)	0.2 (−0.06 to 0.5)	0.13	0.1 (−0.1 to 0.3)	0.39	0.1 (−0.09 to 0.3)	0.25
IN	V	−4.7 (0.3)	−4.9 (0.5)	−4.8 (0.5)	0.01 (−0.2 to 0.2)	0.93	−0.09 (−0.3 to 0.1)	0.45	0.2 (−0.02 to 0.4)	0.08
SN	A	−4.8 (0.3)	−5.2 (1.0)	−4.7 (0.4)	−0.2 (−0.3 to −0.006)	**0.04**	−0.5 (−0.8 to −0.1)	**0.009**	0.3 ( −0.05 to 0.7)	0.09
SN	V	−4.8 (0.6)	−4.9 (0.5)	−4.9 (0.5)	0.02 (−0.2 to 0.3)	0.86	−0.08 (−0.3 to 0.1)	0.50	0.05 (−0.2 to 0.3)	0.74

The model was adjusted for age and sex. Bolded values are statistically significant.

## Discussion

To our knowledge, this pilot study is the first to examine differences in peripheral regions of the retina among Alzheimer's dementia, MCI, and normal cognition using UWF imaging. We observed an increased midperipheral FD, indicating increased branching complexity, in participants with Alzheimer's dementia. Previous studies featuring conventional fundus photographs have found decreased FD in Alzheimer's disease^21^^,^^33^ and cognitive impairment.^26^^,^^27^ Previous work using UWF imaging obtained FD measures from the arteriolar and venular components of the retinal vascular network in Alzheimer's disease (13 participants) compared to cognitively normal controls (14 participants) and reported a significant decrease in arteriolar FD in Alzheimer's disease at baseline, although this was not observed in images captured at a 2-year follow-up (9 AD participants; 14 control participants).^6^ Venular FD has been found to be positively associated with increased cerebral blood flow,^26^^,^^28^ with a reduced venular FD observed in Alzheimer's disease,^29^ whereas a greater decrease in arteriolar network complexity was associated with increased cerebral small-vessel disease pathology^30^^,^^31^ and stroke.^32^ These findings suggest that there may be differential pathological influences on arteriolar and venular networks that our measurements are not currently able to reflect due to the current absence of a reliable automatic procedure for retinal arteriole and venule segmentation on UWF images. We observed a decrease in branching complexity in MCI and an increase in Alzheimer's dementia when compared to cognitively normal controls. There is temporal heterogeneity in the progression of Alzheimer's disease such that it is challenging to identify precisely where on the progression pathway an individual is. It is therefore possible that changes to blood flow may be responding dynamically to pathological stimuli. However, characteristics of previously reported Alzheimer's disease cohorts vary (inclusion and exclusion criteria for participant stratification is not uniform) and is thus likely to be contributing to the variation in results observed. This, in addition to different methodological approaches, poses a challenge in directly comparing our study to previous work.

Although FD is undoubtedly a promising measure of the global integrity of the vascular network, conflicting findings in the literature have been attributed to lack of methodological standardization.^33^ We addressed this by defining a standardized ROI that can be used across UWF datasets to investigate longitudinal changes in vascular branching complexity within this region. Additionally, the accuracy at which vessels can be detected and later quantified introduces an additional source of variation between findings.^20^ We used VAMPIRE software that performs fine vessel segmentation using a deep neural network and may have detected more of the vasculature than in previous studies.

We observed increased rates of arteriolar and venular thinning in participants with Alzheimer's dementia and MCI compared to individuals with normal cognition. In concordance with our findings, the previous study using UWF images reported a significant increase in superotemporal arteriole WG of individuals with Alzheimer's disease at 2-year follow-up that was not observed at baseline.^6^ The effects of an increased rate of thinning may lead to an environment more susceptible to degeneration with limited access to nutrients, decreased oxygenation, and less efficient clearance in the more peripheral retina.

We found limited evidence of differences in tortuosity between the groups. Findings of tortuosity, largely from fundus photography, have been conflicting. Studies have reported decreased venular tortuosity,^21^ decreased arteriolar tortuosity,^29^ and increased arteriolar and venular tortuosity^34^ in Alzheimer's disease. A previous study of tortuosity in UWF images found no differences in tortuosity between Alzheimer's disease and cognitively normal individuals.^6^ However, tortuosity is not invariant to scaling and this should be carefully considered when interpreting our results and comparing them to previous work that uses conventional fundus photography. Tortuosity measured on UWF images has not been widely reported, and we highlight this as interesting area for further technical development and exploration to uncover any relationship between tortuosity and Alzheimer's disease that currently remains uncertain.

Limitations in our study are important to consider. Case sample sizes were relatively small, and there may be unknown confounding factors (e.g., unrecorded medications, vitamin supplements) influencing retinal microvascular variation that have not been controlled for in our analyses. Controlling for various possible confounders may provide a more accurate reflection of any independent effect of dementia pathology on retinal measurements. The lack of associations for some measurements should be treated with a degree of caution as there might have been small but clinically relevant differences between groups that our pilot study was not subsequently powered to detect. Therefore, although these differences are plausible, replication is required.

In addition, it is important to consider the reliability of vascular measures derived from the automatic segmentation of the vasculature. Although manual correction and exclusion of low-quality segmentations were applied to improve the accuracy of the measurements, it is unclear how these measures fluctuate between a participant's image captured at the same time point. To identify signal from noise when performing group comparisons, repeat imaging would be required. The development of the standardized ROI (created to standardize the sampling space of the vasculature for FD computation) could be applied to such a setting and facilitate comparisons cross-sectionally and longitudinally within and between participants. Moreover, we grouped our population based on clinical presentations where associations between retinal changes and neurodegenerative pathology are hypothesized to take place through shared pathological processes. Older people without cognitive impairment, people with MCI, and indeed people with Alzheimer's dementia may all have substantial amyloidosis, cerebrovascular changes, and aggregation of tau. Future studies can look at the association between Alzheimer's disease and retinal changes or direct correlations between the extent of neurodegenerative disease and retinal disease directly, irrespective of the clinical or functional status of the individual.

In our pilot study, we provide some evidence supporting retinal differences in Alzheimer's dementia and MCI with small but statistically significant differences in vessel branching and vessel thinning between those with or at risk of dementia and those who are cognitively normal. Statistical differences from measurements obtained in more peripheral regions of the retina were observed in MCI and Alzheimer's dementia. However, to establish the clinical importance of these differences requires longitudinal group-level between-patient contrasts and within-patient contrasts and therefore could not be established in the pilot study. For example, longitudinal retinal images from individuals with cognitive decline, diabetes, and hypertension (which were exclusion criteria for this pilot study) would be required to assess whether the trends reported here are disease specific. Moreover, although all eligible patients were invited to participate in this pilot study, 90% were Caucasian. Future studies should assess retinal vascular measures from a broad demographic range. Nevertheless, we encourage the use of standardizing measurements on UWF images as presented here as UWF imaging becomes more widely adopted in large-scale studies. Such work would reveal whether the retinal microvasculature as captured using UWF imaging can demonstrate microvascular signs of altered vascular competence and can become a valuable tool for investigating longitudinal changes in MCI and Alzheimer's disease.

## Supplementary Material

Supplement 1
