# Supplementary material for: Retinal Vascular Changes in Alzheimer's Dementia and Mild Cognitive Impairment: A Pilot Study Using Ultra-Widefield Imaging
Source: Transl Vis Sci Technol. 2023 Jan 9;12(1):13. doi: 10.1167/tvst.12.1.13 (PMC9838583; doi:10.1167/tvst.12.1.13)
Supplement: Supplement 1 [file tvst-12-1-13_s001.pdf]

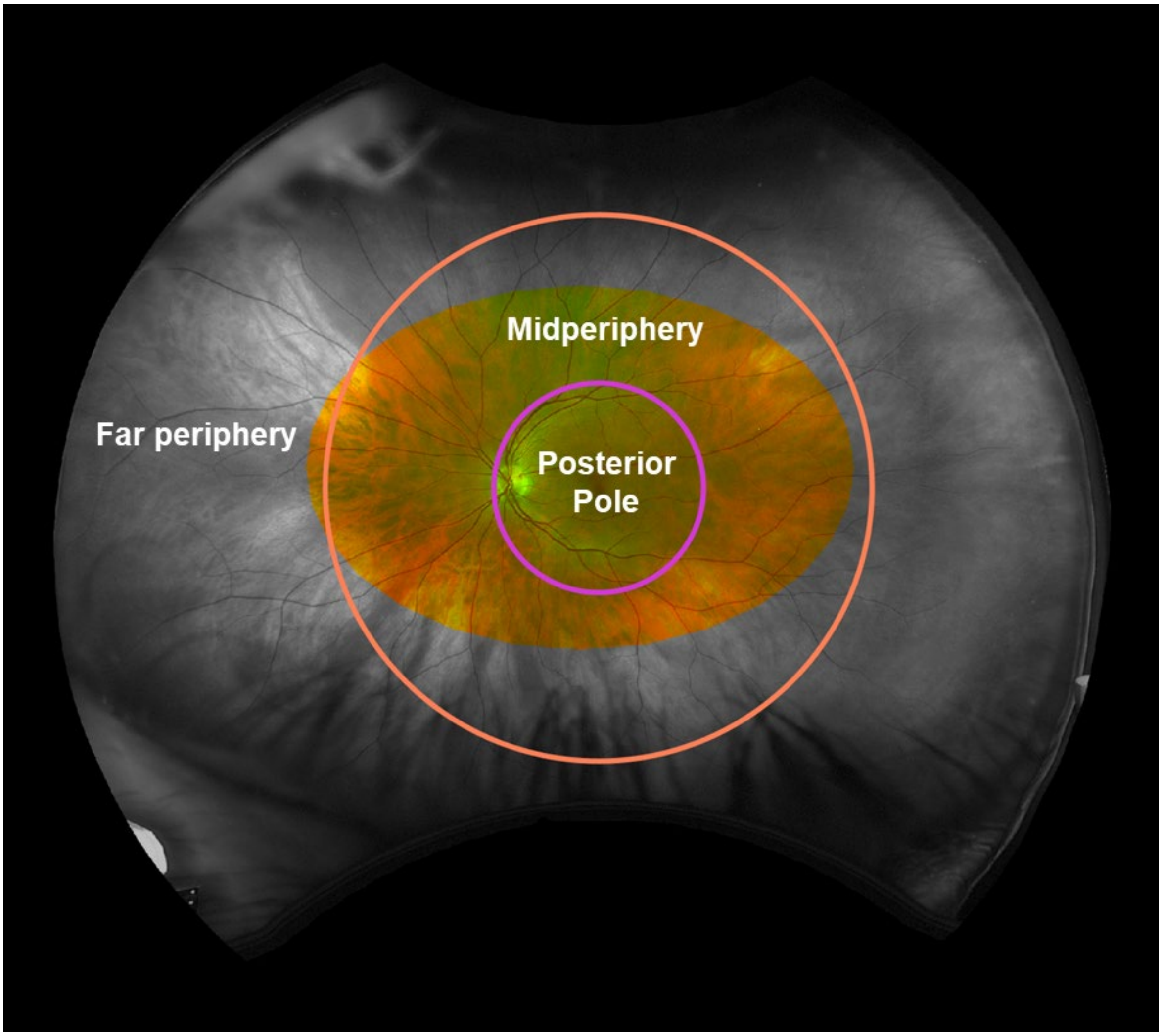

Supplementary Figure S1. Visualisation of the *Standardised* ROI (non-greyscale portion of the image) in the context of imaging modality field of view (FOV) and key regions in the retina. At an approximate 50° FOV (standard fundus photograph imaging device, 5.2mm radius, purple) the optic disc and arcades are captured, representing the posterior pole. At an approximate 120° FOV (widefield imaging device, 12.6mm radius, orange) captures up to the posterior edge of the vortex vein ampulla, capturing the midperiphery. At an approximate 200° FOV (ultra-widefield imaging device, 20.9mm radius) the far periphery is captured. The total area of the *Standardised* ROI is 319mm^2 and includes the posterior pole, some of the midperiphery and a small portion of the far periphery.
